# Targeting the K-Ras - JNK axis eliminates cancer stem-like cells and prevents pancreatic tumor formation

**DOI:** 10.18632/oncotarget.2087

**Published:** 2014-06-10

**Authors:** Masashi Okada, Keita Shibuya, Atsushi Sato, Shizuka Seino, Shuhei Suzuki, Manabu Seino, Chifumi Kitanaka

**Affiliations:** ^1^ Department of Molecular Cancer Science, Yamagata University School of Medicine, Yamagata, Japan; ^2^ Oncology Research Center, Research Institute for Advanced Molecular Epidemiology, Yamagata University, Yamagata, Japan; ^3^ Global COE program for Medical Sciences, Japan Society for Promotion of Science, Tokyo, Japan; ^4^ Department of Neurosurgery, Yamagata University School of Medicine, Yamagata, Japan; ^5^ Research Institute for Promotion of Medical Sciences, Yamagata University School of Medicine, Yamagata, Japan; ^6^ Department of Clinical Oncology, Yamagata University School of Medicine, Yamagata, Japan; ^7^ Department of Obstetrics and Gynecology, Yamagata University School of Medicine, Yamagata, Japan

**Keywords:** pancreatic ductal adenocarcinoma (PDAC), xenograft analysis, tumorigenicity, cancer initiating cell

## Abstract

Cancer cells with self-renewal and tumor-initiating capacity, either quiescent (cancer stem cells, CSCs) or proliferating (cancer stem-like cells, CSLCs), are now deemed responsible for the pervasive therapy resistance of pancreatic cancer, one of the deadliest human cancers characterized by high prevalence of K-Ras mutation. However, to date, much remains unknown how pancreatic CSCs/CSLCs are regulated. Here we show that the K-Ras – JNK axis plays a pivotal role in the maintenance of pancreatic CSCs/CSLCs. *In vitro* inhibition of JNK, either pharmacological or genetic, caused loss of the self-renewal and tumor-initiating capacity of pancreatic CSLCs. Importantly, JNK inhibition *in vivo* via systemic JNK inhibitor administration, which had no discernible effect on the general health status of mice, efficiently depleted the CSC/CSLC population within pre-established pancreatic tumor xenografts. Furthermore, knockdown of K-Ras in pancreatic CSLCs with K-Ras mutation led to downregulation of the JNK pathway as well as in loss of self-renewal and tumor-initiating capacity. Together, our findings suggest that pancreatic CSCs/CSLCs are dependent on K-Ras activation of JNK and also suggest that the K-Ras – JNK axis could be a potential target in CSC/CSLC-directed therapies against pancreatic cancer.

## INTRODUCTION

Pancreatic cancer is one of the leading causes of cancer-related mortality world-wide [1, 2] and is also among the deadliest of all human cancers, with a median survival less than 1 year and no more than one tenth of the patients surviving 5 years [1, 2]. The pervasive resistance of pancreatic cancer against conventional chemoradiotherapy, in combination with the difficulty of early detection, is considered to be the major contributing factor to the highly dismal prognosis of this devastating disease [3-5]. Although tremendous efforts have therefore been made for the past decades to overcome the therapy resistance of pancreatic cancer, strikingly, the mortality from pancreatic cancer is still on the rise in sharp contrast to the all cancer mortality, which has been declining steadily since the early 1990s [2]. Obviously, there is a dire need for novel and innovative approaches based on ground-breaking ideas to tackle this intractable malignancy.

Since the identification of cancer stem cells (CSCs) or cancer stem-like cells (CSLCs) in pancreatic cancer [6, 7], pancreatic CSCs/CSLCs have emerged as a possible, attractive explanation for the highly incorrigible therapy resistance of pancreatic cancer [8-12]. CSCs and CSLCs, which are distinguished according to whether they are quiescent or proliferating [13, 14], are subpopulations of tumor cells characterized by unlimited self-renewal and tumor-initiating capacity, and are often associated with high therapy resistance [13-16]. Not surprisingly, the molecular mechanism contributing to the maintenance of pancreatic CSCs/CSLSc have been an intense focus of recent research, with an enthusiastic expectation that elucidation of the mechanism would lead to development of effective measures to target pancreatic CSCs/CSLCs and by so doing to substantial improvement in the prognosis of pancreatic cancer. So far, a number of molecules and signaling pathways have been identified and implicated in the mechanism [8-12], however, much still remains to be learned as to how we can efficiently eliminate CSCs/CSLCs from tumors of pancreatic cancer, and that, in therapeutically feasible and significant manners.

The c-Jun NH_2_-terminal kinases (JNKs), a subgroup of mitogen-activated protein kinases, are often deregulated in a variety of human cancers including pancreatic cancer, although the role and significance of JNK deregulation in such human cancers have not been fully delineated [17-21]. Recently, we and others have shown *in vitro* that JNK is required for the maintenance of the self-renewal and tumor-initiating capacity of human glioblastoma CSLCs, giving rise to the novel possibility that the maintenance of cancer stem (-like)/initiating cells may be one of the critical roles of JNK in human cancers [22-24]. Most importantly, we have further demonstrated using preclinical animal models that systemic JNK inhibitor administration to tumor-bearing mice eliminates the CSC/CSLC population within the tumors effectively and safely, suggesting that JNK not only plays an essential role in the maintenance of glioblastoma CSCs/CSLCs *in vivo* but also could serve as a practical therapeutic target for cancers whose stem (-like) cells are dependent on JNK [22, 24]. However, to date, the role of JNK in the CSCs/CSLCs of human cancers other than glioblastoma remains unexplored.

Here in this study, we discovered that the JNK-dependent mechanism of CSC/CSLC maintenance is operative in human pancreatic cancer and demonstrated that the mechanism can be targeted safely and effectively *in vivo*. Furthermore, we also provide evidence that K-Ras contributes to the maintenance of pancreatic CSCs/CSLCs through JNK activation.

## RESULTS

### JNK activity is essential for the maintenance of the self-renewal and tumor-initiating capacity of pancreatic CSLCs *in vitro*

**Figure 1:**
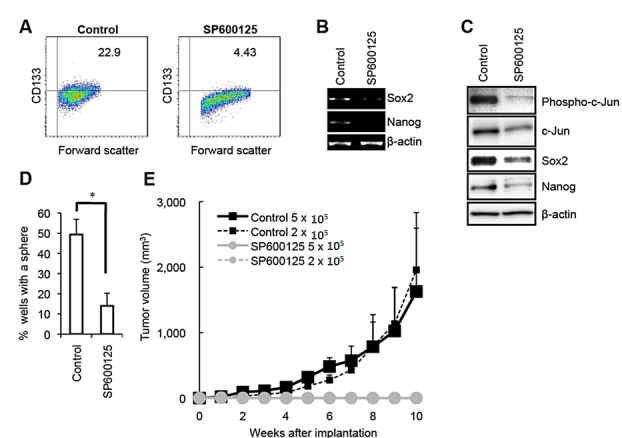
Pharmacological inhibition of JNK by SP600125 causes loss of the self-renewal and tumor-initiating capacity in PANC-1 CSLCs (A) PANC-1 CSLCs cultured in the absence (Control) or presence of 30 μM SP600125 for 6 days were subjected to flow cytometric analysis for the cell surface expression of CD133. Representative flow cytometric plots together with the percentages of CD133-positive cells are shown. (B and C) PANC-1 CSLCs cultured as in (A) were subjected to RT-PCR (B) and immunoblot (C) analyses of the indicated mRNAs or proteins. (D) PANC-1 CSLCs cultured as in (A) were subjected, after washout of the inhibitor, to the sphere formation assay in the absence of SP600125. The graph shows the percentage of wells in which a tumorsphere has been formed from a single cell, and the data represent means + SD from 3 independent experiments. (E) Mice (3 for each group) were implanted subcutaneously with the indicated numbers of viable PANC-1 CSLCs that had been treated with or without SP600125 as in (A). Tumor volumes were measured at the indicated time points after implantation, and the results are presented in the graph as the means + SD.

**Figure 2:**
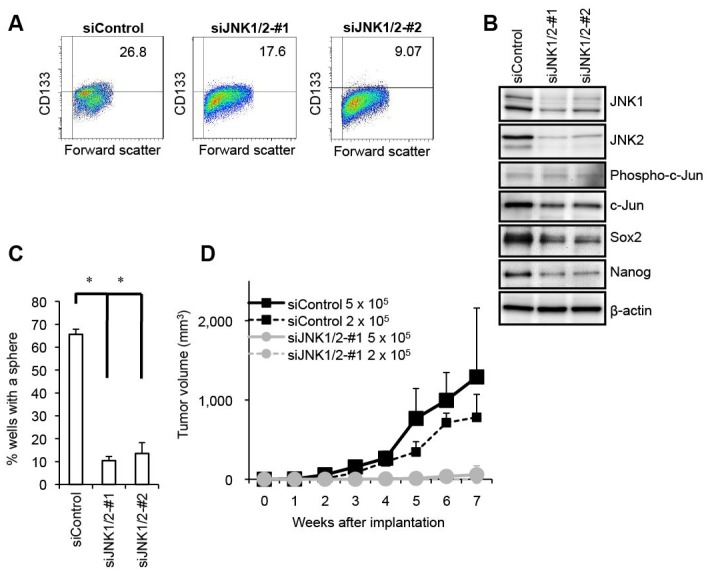
Genetic silencing of JNK by siRNA causes loss of the self-renewal and tumor-initiating capacity in PANC-1 CSLCs (A) PANC-1 CSLCs were transiently transfected with 2 combinations (#1 and #2) of siRNAs against JNK1 and JNK2 (siJNK1/2) or with a control siRNA (siControl), as detailed in Materials and methods. After 8 days, the transfected cells were subjected to flow cytometric analysis for the cell surface expression of CD133. Representative flow cytometric plots together with the percentages of CD133-positive cells are shown. (B) PANC-1 CSLCs treated as in (A) were subjected to immunoblot analysis for the expression of the indicated proteins. (C) PANC-1 CSLCs treated as in (A) were subjected to the sphere formation assay. The graph shows the percentage of wells in which a tumorsphere has been formed from a single cell, and the data represent means + SD from 3 independent experiments. (D) Mice (3 for each group) were implanted subcutaneously with the indicated numbers of viable PANC-1 CSLCs that had been treated as in (A). Tumor volumes were measured at the indicated time points after implantation, and the results are presented in the graph as the means + SD.

To explore the mechanism underlying the maintenance of pancreatic CSLCs, we first isolated sphere-forming cells from PANC-1 xenograft tumors, which are reportedly enriched for pancreatic cancer cells with self-renewal and tumor-initiating capacity [25-28]. Consistent with the earlier reports, the sphere-forming cells from PANC-1 xenografts (hereafter termed PANC-1 CSLCs) were enriched for cells with surface expression of CD133, a known marker for CSCs/CSLCs of a variety of human cancers including pancreatic cancer (Supplementary Figure S1A) [29, 30] and expressed higher levels of pluripotency markers such as Sox2 and Nanog (Supplementary Figure S1B,), as compared with their differentiated counterparts. Sphere formation and xenograft assays also demonstrated PANC-1 CSLCs have self-renewing and tumor-initiating capacity that are lost upon differentiation (Supplementary Figure S1D, E). Using PANC-1 CSLCs as a model, we then set out to search for molecules or pathways that are differentially expressed and/or activated in self-renewing and differentiated PANC-1 CSLCs. Intriguingly, just as we observed with CSLCs of glioblastoma [22], we noted during the course of our experiments that the activity of the JNK pathway, as represented by the expression of phosphorylated JNK and c-Jun, was higher in self-renewing cells than in their differentiated counterparts (Supplementary Figure S1C). We therefore wished to determine whether JNK has a role in the control of PANC-1 CSLCs similarly to glioblastoma CSLCs. Treatment of PANC-1 CSLCs with a pharmacological inhibitor SP600125 at 30 μM, which effectively inhibited the JNK activity of PANC-1 CSLCs (Figure 1C) without significantly compromising their viability (Supplementary Figure S2), reduced the proportion of CD133-positive cells as well as the expression levels of Sox2 and Nanog (Figure 1A - C). To determine whether the observed changes in the stem cell marker expression reflected loss of their self-renewal capacity, PANC-1 CSLCs pretreated with SP600125 were tested for their ability to form spheres in the absence of SP600125. The results indicated PANC-1 CSLCs had substantially lost their sphere-forming ability after the transient SP600125 pretreatment (Figure 1D), suggesting that JNK is required for the maintenance of the self-renewal capacity of PANC-1 CSLCs. To ascertain that the effect of SP600125 on PANC-1 CSLCs was via inhibition of JNK, we next examined the effect of JNK knockdown on their stem cell marker expression and sphere-forming ability. To this end, we transiently transfected into PANC-1 CSLCs combinations of siRNAs directed against *JNK1* and *JNK2*, which decreased the expression of JNK1 and JNK2 (Figure 2B). Under this JNK knockdown condition, the proportion of CD133-positive cells, the expression of Sox2 and Nanog, and the sphere-forming ability were significantly decreased in JNK knockdown cells as compared to the control cells (Figure 2A - C). Thus, the results of the JNK inhibition experiments, both pharmacological and genetic, consistently suggested that JNK plays a critical role in the maintenance of the self-renewal capacity of PANC-1 CSLCs. We then wished to ask if this role of JNK in CSLC maintenance is shared among pancreatic CSLCs. To this end, we isolated sphere-forming tumor cells from PSN-1 pancreatic cancer xenografts (PSN-1 CSLCs) (Supplementary Figure S3) and investigated the role of JNK in PSN-1 CSLCs. The results indicated that both pharmacological and genetic JNK inhibition caused significant loss of the stem cell marker expression and sphere-forming ability of PSN-1 CSLCs (Supplementary Figures S4 and S5), essentially similarly to PANC-1 CSLCs. Together, the data demonstrate that JNK plays an essential role in the maintenance of the self-renewal capacity of *in vitro* cultured pancreatic CSLCs.

Along with the self-renewal capacity, the tumor-initiating capacity is one of the key properties of CSCs/CSLCs that distinguish themselves from non-stem cancer cells. We therefore determined next whether JNK is also required for the maintenance of the tumor-initiating capacity of pancreatic CSLCs. When different numbers of PANC-1 CSLCs with or without SP600125 pretreatment were implanted into nude mice, we found that 2 × 10^5^ control PANC-1 CSLCs were sufficient to initiate subcutaneous tumors whereas no tumors were initiated by implantation of as many as 5 × 10^5^ PANC-1 CSLCs pretreated with SP600125 (Figure 1E). Essentially similar results were obtained from knockdown experiments, where JNK knockdown cells showed significantly compromised tumor-initiating capacity in the xenograft analysis as compared to the control knockdown cells (Figure 2D). Thus, the data suggest that JNK is required for the maintenance of the tumor-initiating capacity of *in vitro* cultured pancreatic CSLCs.

### JNK activity is required for the maintenance of the pancreatic CSC/CSLC population within a tumor but not for bulk tumor growth *in vivo*

**Figure 3:**
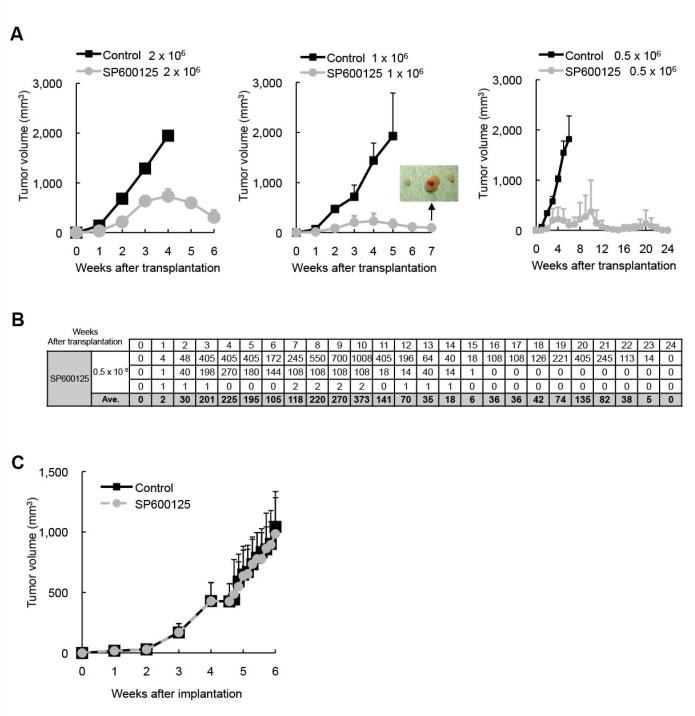
Transient JNK inhibition *in vivo* depletes pancreatic CSCs/CSLCs within tumors without having an immediate inhibitory effect on bulk tumor growth Mice implanted subcutaneously with PANC-1 CSLCs (1x10^6^ viable cells) were randomized into 2 treatment groups (8 mice per group) at 4 weeks after implantation when the average tumor volume reached ~ 400 mm^3^, and received a daily intraperitoneal injection of the control vehicle or SP600125 (60 mg/kg/day) for 10 consecutive days. On the next day of the final drug treatment, the subcutaneous tumors (primary tumors) were excised and dissociated, and serial dilutions of the dissociated tumor cells were transplanted subcutaneously into new mice. (A) The volumes of the secondary tumors (n = 3 for each group) formed by transplantation of the indicated numbers (*Left*: 2 × 10^6^, *Middle*: 1 × 10^6^, *Right*: 0.5 × 10^6^) of viable cells from the primary tumors treated without (Control) or with SP600125 are presented as the means + SD in the graphs. The inset in the graph for 1x10^6^-cell transplantation demonstrates the presence of subcutaneous tumors at the end of the observation period, when the mice with actively regressing subcutaneous masses formed by transplantation of SP600125-treated tumor cells were sacrificed for confirmation of tumor presence. (B) The volumes (in mm^3^) of the secondary tumors formed by transplantation of 0.5 × 10^6^ cells from SP600125-treated primary tumors (n = 3) are also shown in the table separately from the graph in (A). (C) The volume of the primary tumors treated without (Control) or with SP600125 was assessed at the indicated time points and is presented in the graph as the mean + SD.

Our *in vitro* analyses conducted thus far indicated that JNK activity is required for the maintenance of pancreatic CSLCs, at least in a defined, artificial cell culture condition. To determine whether such *in vitro* findings are relevant to CSCs/CSLCs in a more natural, pathophysiological condition, we examined by the serial transplantation assay the impact of JNK inhibition *in vivo* on CSCs/CSLCs residing in pre-established tumors. Mice harboring subcutaneous tumors formed by implantation of PANC-1 CSLCs (primary tumors) were transiently treated with or without systemic administration of SP600125 (60 mg/kg/day) for 10 consecutive days, after which the subcutaneous tumors were excised and dissociated tumor cells were transplanted subcutaneously into new mice after serial dilution (2 × 10^6^, 1 × 10^6^, 0.5 × 10^6^). When the mice were then monitored for the development and growth of secondary tumors, all mice transplanted with cells from the control-treated tumors developed secondary tumors that grew steadily to become larger than 2,000 mm^3^ (Figure 3A). Meanwhile, most of the mice transplanted with cells from the SP600125-treated tumors did develop tumors, at least initially. However, quite strikingly, the secondary tumors derived from the SP600125-treated primary tumors started to regress spontaneously ~ 4 weeks after transplantation (Figure 3A), suggesting that JNK inhibition *in vivo* limited, albeit did not abolish immediately, the self-renewal capacity of pancreatic CSCs/CSLCs to such a degree that they could no longer perpetuate tumor growth. In support of this idea, when some of the regressing tumors were subjected to flow cytometric analysis for CD133 expression, we found that the regressing secondary tumors contained significantly fewer, though not negligible, CD133-positive cells than the steadily growing secondary tumors (i.e., derived from the control-treated primary tumors) (Supplementary Figure S6). Extended observation of the remaining secondary tumors derived from the SP600125-treated primary tumors confirmed that all regressing tumors eventually became invisible and impalpable, i.e., regressed completely. Curious enough, the growth curve for one secondary tumor showed a tri-phasic pattern (= three rounds of growth coupled with regression), illuminating the possibility that the primary tumor had contained “delayed contributing” tumor-initiating cells [31] whose self-renewing capacity was limited by SP600125 treatment (Figure 3A,). Notably, in contrast to the marked inhibitory effect of *in vivo* JNK inhibition on the CSC/CSLC population within the tumors, the exactly identical SP600125 treatment protocol failed to have the slightest inhibitory effect on bulk tumor growth during the 10-day treatment period (Figure 3C). Collectively, our results suggest that, *in vivo*, JNK activity may be specifically required for the maintenance of CSCs/CSLCs but not for the proliferation and survival of non- CSCs/CSLCs and that JNK inhibition may set a limit to the otherwise limitless self-renewal capacity of pancreatic CSCs/CSLCs.

### K-Ras knockdown results in downregulation of JNK signaling and phenocopies the effect of JNK inhibition on pancreatic CSLCs

**Figure 4:**
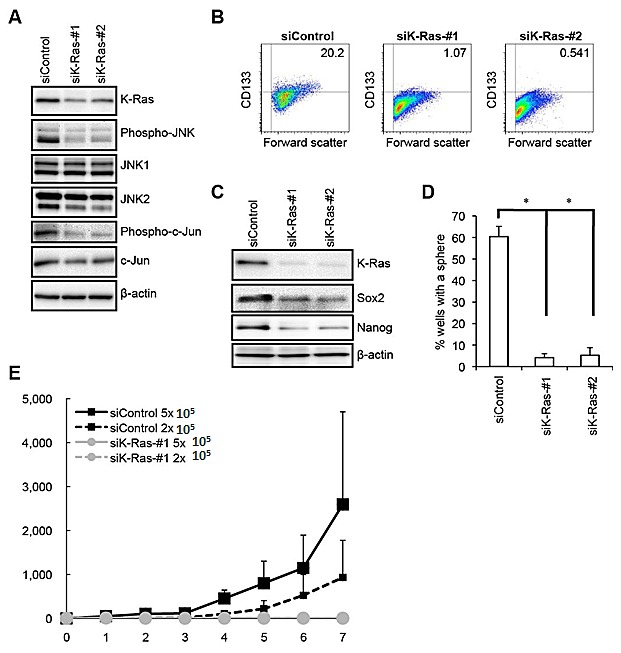
K-Ras is required for the maintenance of the JNK pathway activity as well as for the maintenance of the self-renewal and tumor-initiating capacity of PANC-1 CSLCs (A) PANC-1 CSLCs transiently transfected with siRNAs against K-Ras (siK-Ras) or with a control siRNA (siControl) were subjected to immunoblot analysis of the indicated proteins at 4 days after transfection, to monitor K-Ras expression as well as the activation status of the JNK pathway. (B) PANC-1 CSLCs transiently transfected with siRNAs against K-Ras (siK-Ras) or with a control siRNA (siControl) for 8 days were subjected to flow cytometric analysis to examine the cell surface expression of CD133. Representative flow cytometric plots together with the percentages of CD133-positive cells are shown. (C) PANC-1 CSLCs treated as in (B) were subjected to immunoblot analysis for the expression of K-Ras and the indicated stem cell markers. (D) PANC-1 CSLCs treated as in (B) were subjected to the sphere formation assay. The graph shows the percentage of wells in which a tumorsphere has been formed from a single cell, and the data represent means + SD from 3 independent experiments. (E) Mice (3 for each group) were implanted subcutaneously with the indicated numbers of viable PANC-1 CSLCs that had been treated as in (B). Tumor volumes were measured at the indicated time points after implantation, and the results are presented in the graph as the means + SD.

Having demonstrated the critical role of JNK in the maintenance of pancreatic CSCs/CSLCs *in vitro* and *in vivo*, we next turned our attention to the mechanism by which the JNK activity of pancreatic CSCs/CSLCs is maintained. Since JNK was originally identified as a kinase activated by Ras [32, 33], and given that K-Ras is mutationally activated in more than 90% of pancreatic ductal adenocarcinoma [34, 35], we first sought to determine whether K-Ras has a role in the control of JNK signaling in pancreatic CSCs/CSLCs. To do this, we knocked down using two different siRNAs the expression of *K-Ras* in PANC-1 CSLCs, which were derived from PANC-1 cells having a mutated (G12D) *K-Ras* allele [36]. The results of knockdown experiments indicated that both siRNAs directed against *K-Ras* caused reduced phosphorylation of JNK as well as that of c-Jun (Figure 4A), suggesting that K-Ras expression is required for the maintenance of JNK signaling in PANC-1 CSLCs. Given that K-Ras is required for the JNK activity which we have just demonstrated to play an essential role in the maintenance of pancreatic CSLCs, we next went on to ask if K-Ras also has a role in the maintenance of PANC-1 CSLCs. The results of the stem cell marker analyses indicated that *K-Ras* knockdown reduced the proportion of CD133-positive cells, as well as the expression of Sox2 and Nanog in PANC-1 CSLCs (Figure 4B,). *K-Ras* knockdown also inhibited the sphere-forming ability of PANC-1 CSLCs (Figure 4D), in good agreement with the results of the stem cell marker analyses. Together, these data suggested that K-Ras activation of JNK plays a key role in the maintenance of the self-renewal capacity of PANC-1 CSLCs. We next determined whether this role of the K-Ras – JNK axis is unique to PANC-1 CSLCs or is commonly shared by other pancreatic CSLCs by examining PSN-1 cells, which are also reported to harbor a mutation (G12R) in the *K-Ras* gene [37]. The results of the *K-Ras* knockdown experiments using PSN-1 CSLCs were essentially similar to those obtained from PANC-1 CSLCs (Supplementary Figure S7), in favor of the idea that the K-Ras – JNK axis may have a common role in the maintenance of the self-renewal capacity of pancreatic CSLCs. Finally, to confirm that K-Ras also contributes to the maintenance of the tumor-initiating capacity of pancreatic CSCs/CSLCs, we tested the tumor-initiating capacity of *K-Ras* knockdown cells in the xenograft analysis. Whereas implantation of 2 × 10^5^ cells was sufficient for control knockdown cells to initiate steadily growing tumors, *K-Ras* knockdown PANC-1 CSLCs failed to initiate tumor formation throughout the extended observation period even when as many as 5 × 10^5^ cells were implanted (Figure 4E). Combined, these data suggest that K-Ras activation of JNK has a critical role in the maintenance of the self-renewal and tumor-initiating capacity of pancreatic CSCs/CSLCs.

## DISCUSSION

Pancreatic cancer is one of the most intractable of all human cancers with high therapeutic resistance, and currently, pancreatic CSCs/CSLCs are drawing increasing and considerable attention as a possible culprit for the therapeutic resistance of pancreatic cancer [8-12]. So far, with an intention to identify novel therapeutic targets to overcome the therapy resistance, efforts have been made to dissect the molecular pathways involved in the control of pancreatic CSCs/CSLCs, which has led to the identification of a number of pathways implicated in their control. For instance, the Hedgehog [38-40], Notch [41], and Nodal/Activin [26] pathways have been identified as having a role in the maintenance of pancreatic CSCs/CSLCs, for which pharmacological agents modulating their activities are already available for use and have shown therapeutic effects in preclinical models of pancreatic cancer. While such pharmacological agents are thus expected to make attractive candidates to be tested in the clinical settings in future, apparently, information on targetable pathways/molecules for the control of pancreatic CSCs/CSLCs is still absolutely limited. In this study, therefore, we attempted to expand our knowledge on the molecular mechanisms by which pancreatic CSCs/CSLCs are maintained. Our findings indicated that inhibition of JNK in pancreatic CSLCs resulted in decreased stem cell marker expression and sphere-forming ability, as well as in loss of their tumor-initiating capacity, thus identifying JNK as a hitherto unrecognized, novel key player in the control of pancreatic CSCs/CSLCs.

As one of the most important findings of this study, we have demonstrated by the serial transplantation assay that systemically-administered JNK inhibitor quite efficiently deprives CSCs/CSLCs of their capacity to sustain tumor growth *in vivo*. Given that the serial transplantation assay, coupled with serial dilution of the cells to be transplanted, is currently the most reliable, albeit not flawless, method to evaluate the incidence of CSCs/CSLCs [15, 42], our results make out a strong case for the idea that JNK is essential for the maintenance of pancreatic CSCs/CSLCs *in vivo*. Furthermore, we confirmed that the protocol of JNK inhibitor treatment we adopted for the serial transplantation assay of this study (60 mg/kg/day for 10 consecutive days) was well tolerated by nude mice and did not affect their general health status for at least 2 months (Supplementary Figure S8). Combined, these findings suggest that JNK is a promising target molecule to eliminate pancreatic CSCs/CSLCs *in vivo*.

Significantly, transplantation of tumor cells from SP600125-treated primary tumors once led to formation of secondary tumors in most of the recipient mice, all of which eventually regressed spontaneously. This unique observation provides unequivocal, important evidence that JNK inhibition *in vivo* did not critically interfere with the process of tumor cell “engraftment” per se, which is an artificial process that inevitably accompanies and is inherent in xenograft analyses. Instead, the observation could more plausibly be explained by the idea that JNK inhibition *in vivo* turned long-term tumor-initiating cells (*bona fide* CSCs/CSLCs) into tumor transient amplifying cells which are assumed to have limited or no self-renewal capacity and contribute to tumor formation only in primary mice [31]. We speculate that ultimate depletion of CSCs/CSLCs took place at some time point in the course of the secondary tumor formation, which led to cessation of cell supply and tipped the balance of the tumor tissue kinetics in favor of regression, i.e., more cell loss than cell gain. In one recipient mouse, we observed 3 rounds of tumor formation and regression, the latest of which occurred around 4 – 5 months after transplantation. This finding may lend support to the idea that there existed delayed contributing tumor-initiating cells (which are also *bona fide* CSCs/CSLCs) in the primary tumors, the self-renewal capacity of which was limited (turned into tumor transient amplifying cells) by JNK inhibition. Importantly, for as long as 6 months, no sustained tumor growth has taken place in any of the mice transplanted with tumor cells from SP600125-treated primary tumors (Figure 3A and B), which implies that the short-term, transient JNK inhibition was sufficient to deprive CSCs/CSLCs of their tumor-initiating capacity and at the same time prevent them from recovering it *in vivo* at least during the observation period. Although it is practically impossible to test whether or not the effect of JNK inhibition is actually “irreversible” using xenograft models involving an experimental animal with relatively short life span, our results suggest that JNK inhibition may be a promising approach to achieve long-term control over pancreatic CSCs/CSLCs.

Notably, the exactly identical treatment protocol that so effectively eliminated CSCs/CSLCs within the primary tumors had virtually no inhibitory effect on the growth of the primary tumors during the 10-day treatment period, implying that JNK activity is “not immediately required” for pancreatic cancer cells to maintain proliferation and survival. A similar pattern was reported in a previous study in which the effect of systemic administration of a Hedgehog inhibitor was tested against pancreatic tumor xenografts [43]. Our results, in conjunction with those reported in the study, thus underscore the potential difficulty in proper evaluation of the anti-tumor effect of CSC/CSLC-specific drugs. Although our treatment protocol failed to control the growth of established tumors, it does not necessarily imply that JNK inhibition is ineffective against bulk pancreatic tumors *in vivo* in any condition, since a recent report clearly demonstrated that the growth (assessed by tumor area) and the tumor cell proliferation (assessed by proliferating cell nuclear antigen positivity) of pancreatic tumors developing in K-Ras^*G12D*^ + Tgfbr2^KO^ mice were significantly inhibited by systemic administration of JNK inhibitors [21]. Nevertheless, given the at best modest inhibitory effects of JNK inhibition on bulk tumor growth shown in the study [21] and in the present study, apparently, it is essential that future JNK-targeting therapies against pancreatic cancers be combined with other treatment modalities aimed at the tumor bulk.

We also demonstrated as another key finding of this study that K-Ras plays an essential role in the maintenance of both the JNK activity in pancreatic CSLCs and their self-renewal and tumor-initiating capacity. The *K-Ras* gene is the most frequently mutated among known oncogenes and is indeed mutated (and activated by mutation) in more than 90% of pancreatic cancers [34, 35]. JNK was originally identified as a kinase activated by H-Ras [32, 33], but subsequently, it was demonstrated that mutated K-Ras activates JNK *in vitro* and *in vivo* [44, 45]. Intriguingly, the *K-Ras*^*G12D*^ mutant, the most common mutant allele in pancreatic cancers [46], has been shown to preferentially activate the JNK pathway rather than the ERK pathway [44]. In line with these findings, a recent study demonstrated that JNK is activated in human pancreatic cancer tissues as well as in pancreatic cancer cell lines, pointing to the possibility that K-Ras may have a role in JNK activation in pancreatic cancers [21]. In addition to such a role of K-Ras mutation in JNK activation, the role of mutated K-Ras in tumor maintenance has also been documented in lung and pancreatic cancer models [47, 48]. In these models, tumor regression caused by K-Ras inactivation was accompanied by extensive apoptosis of the tumor cells [47, 48], suggesting that K-Ras contributes to tumor maintenance at least in part through survival of bulk tumor cells. Significantly, a previous report demonstrated that, in lung and pancreatic cancer cells harboring K-Ras mutation, cells with a well-differentiated epithelial phenotype were more dependent on K-Ras for their viability than those with a less well-differentiated, mesenchymal phenotype [49]. Together, these previous findings suggest that K-Ras mutation may have a role in JNK activation and cellular survival at least in bulk tumor cells of pancreatic cancers with mutated K-Ras. However, in contrast to its well-defined roles in bulk tumor cells [50], the role of Ras mutation in CSCs/CSLCs of K-Ras-mutated tumors has remained unknown so far. Here in this study, we have delineated the role of K-Ras mutation in CSCs/CSLCs using pancreatic CSLCs as a model. Although exogenous expression of mutationally activated K-Ras alleles has been shown in other studies to favor expansion of the CSLC population in colon cancer models [51, 52], to our knowledge, this is the first study to demonstrate the essential role of endogenously expressed K-Ras in the maintenance of the self-renewal and tumor-initiating capacity of CSCs/CSLCs.

Our demonstration in the present study that K-Ras activated JNK in pancreatic CSLCs and that K-Ras as well as JNK was similarly required for their maintenance suggests that the K-Ras – JNK axis plays a pivotal role in the maintenance of the CSC/CSLC population of pancreatic cancer. However, our data do not necessarily exclude the possibilities that K-Ras and JNK have other downstream mediators and upstream activators involved in the control of CSCs/CSLCs, respectively. In addition, although Rac1 is an attractive candidate molecule linking K-Ras and JNK [53], it still remains to be delineated in this study how K-Ras activates JNK in CSLCs. Apparently, unraveling the molecular interplay involved in the control of CSCs/CSLCs by the K-Ras - JNK axis in future studies is expected to provide useful information in identifying novel molecular targets for CSC/CSLC-directed therapies. Of note, beyond pancreatic cancer, an essential role of JNK has been documented in K-Ras-induced lung tumor development [53], which points to the possibility that the role of the K-Ras - JNK axis in the control of CSCs/CSLCs may not be unique to pancreatic cancer but may be shared by other human cancers. In further support of this possibility, we have recently demonstrated that JNK is essential for the maintenance of the tumor-initiating capacity but not for the bulk tumor growth of A549 lung adenocarcinoma cells known to harbor an activating K-Ras mutation [54-56]. It would therefore be of great interest to examine whether the K-Ras – JNK axis has a similar, CSC/CSLC-specific role in other human cancers with mutated Ras. Such an attempt would have great therapeutic impact given the high prevalence of Ras mutation in human cancer.

In conclusion, we have presented for the first time in this study evidence that the K-Ras - JNK axis plays a key role in the maintenance of pancreatic CSCs/CSLCs. Our findings suggest that K-Ras mutation could contribute to tumor development and maintenance at least in part through the maintenance of CSCs/CSLCs, thus providing a novel and significant insight into the role of Ras mutation in tumor cell biology. Furthermore, our data demonstrated that JNK could be a viable target in CSC/CSLC-directed therapies against pancreatic cancer. Combination therapies targeting both the K-Ras - JNK axis in CSCs/CSLCs and the bulk tumor component, for instance by conventional chemotherapies, would therefore be a rational and promising approach to treat pancreatic cancers and hopefully other intractable human cancers with Ras mutation.

## MATERIALS AND METHODS

### Antibodies and reagents

Anti-c-Jun (#9165), anti-phospho-c-Jun (#9261), anti-phospho-JNK (#9251), anti-Sox2 (#3579), anti-Nanog (#4903), anti-E-cadherin (#3195) antibodies were purchased from Cell Signaling Technology, Inc. (Beverly, MA, USA). Anti-β-actin (A1978) was from Sigma (St. Luis, MO, USA). Anti-JNK1 (sc-474), anti-JNK2 (sc-7345) and anti-K-Ras (sc-30) were from Santa Cruz Biotechnology, Inc. (Santa Cruz, CA, USA). Anti-CD133 (W6B3C1) was from Miltenyi Biotech (Germany). SP600125 was purchased from Calbiochem (La Jolla, CA, USA) and was dissolved in DMSO to prepare a 50 mM stock solution.

### Cell culture

The human pancreas cancer cell line PANC-1 was obtained from Cell Resource Center for Biomedical Research, Institute of Development, Aging and Cancer, Tohoku University. PSN-1 was a kind gift from Dr. Teruhiko Yoshida at National Cancer Center Research Institute, who originally established the cell line from pancreatic adenocarcinoma tissue [37]. These cell lines were maintained in DMEM/F12 supplemented with 10% fetal bovine serum (FBS) and 100 units/mL penicillin and 100 μg/mL streptomycin. To establish PANC-1 CSLCs, PANC-1 cells were implanted subcutaneously into nude mice after being cultured and amplified under the monolayer stem cell culture condition [22, 57, 58]. After formation of subcutaneous tumors, the tumors were excised and cells from the tumors were, after dissociation, cultured on non-coated dishes in the stem cell culture medium. Cells from spheres formed under this culture condition were transferred and amplified under the monolayer stem cell culture condition, which were then used as PANC-1 CSLCs after characterization as shown in Supplementary Figure S1. PSN-1 CSLCs were established in a similar manner to PANC-1 CSLCs. The authenticity of PANC-1 CSLCs and PSN-1 CSLCs as cells derived from PANC-1 and PSN-1, respectively, was verified by genotyping of short tandem repeat (STR) loci (Bio-Synthesis, Inc., Lewisville, TX, USA) followed by comparison to the ATCC STR database for Human Cell Lines. Unless otherwise indicated, these pancreatic CSLCs were stably maintained and used for experiments under the monolayer stem cell culture condition, as previously described [22, 58]. In brief, cells were cultured on collagen-I-coated dishes (IWAKI, Tokyo, Japan) in the stem cell culture medium (DMEM/F12 medium supplemented with 1% B27 [Gibco-BRL, Carlsbad, CA, USA], 20 ng/mL EGF and FGF2 [Peprotech, Inc.,Rocky Hill, NJ, USA], D-(+)-glucose [final concentration, 26.2 mM], L-glutamine [final concentration, 4.5 mM], 100 units/mL penicillin and 100 μg/mL streptomycin). In principle, the stem cell culture medium was changed every 3 days, and EGF and FGF2 were added to the culture medium every day. Differentiation of pancreatic CSLCs was induced by culturing the cells under the differentiation-inducing condition (DMEM/F12 containing 10% FBS) for 2 weeks. Throughout the study, the cell number was determined using a hemocytometer, and the cell viability was examined by the dye exclusion method (0.2% trypan blue). Cell viability (%) was defined as 100 × ‘the number of viable cells’/ (‘the number of viable cells’ + ‘the number of dead cells’).

### Gene silencing by siRNA

siRNAs against human JNK1 (#1: VHS40722, #2: VHS40724), JNK2 (#1: VHS40726, #2: VHS40729), and K-Ras (#1: HSS180200, #2: HSS105872) as well as Medium GC Duplex #2 of Stealth RNAi™ siRNA Negative Control Duplexes (as a control for siRNA experiments) were purchased from Invitrogen Life Technologies (Carlsbad, CA, USA). Transfection of siRNAs was performed using Lipofectamine RNAiMAX (Life Technologies) according to the manufacturer's instructions. To achieve sustained knockdown of the target genes, siRNA transfection was repeated 4 days after the initial transfection.

### Sphere formation assay

After being dissociated into single cells, PANC-1 cells were serially diluted in the stem cell culture medium and seeded into non-coated 96-well plates so that there be a single cell in each well. Wells containing a single cell were marked on the next day and, 1 week after seeding, the percentage of marked wells with a sphere relative to the total number of marked wells was determined. For PSN-1 CSLCs, dissociated cells were suspended in the stem cell culture medium at a density of 5 × 10^3^ cells/mL. Then, 2 mL of the cell suspension (1 × 10^4^ cells) was transferred to each well of a non-coated 12-well plate. The number of spheres in each well was counted 5 days after seeding.

### Flow cytometric analysis

Dissociated cells were washed with ice-cold phosphate-buffered saline (PBS), fixed with 4% (w/v) paraformaldehyde for 10 min at room temperature (RT), and washed again with PBS. The cells were then blocked in FACS buffer (0.5% [w/v] bovine serum albumin, 0.1% [w/v] NaN_3_ in PBS) for 30 min, followed by 3 PBS rinses and subsequently by incubation with the anti-CD133 antibody in the FACS buffer for 1 h and then with the Alexa Fluor® 488 goat anti-mouse IgG for another 30 min at RT. Gating for single cells was established using forward scatter in the isotype control samples. The isotype control samples were used to establish a gate in the fluorescein isothiocyanate (FITC) channel. Cells showing signal for CD133 above the gate established by the isotype control were deemed CD133-positive. All flow cytometric analysis experiments were run on FACSCanto™ II Flow Cytometer (BD Biosciences, Franklin Lakes, NJ, USA).

### RT-PCR analysis

Total RNA was extracted with TRIzol (Life Technologies). Total RNA was reverse-transcribed into cDNA using PrimeScriptTM 1^st^ strand cDNA Synthesis kit (Takara, Tokyo, Japan) according to the manufacturer's instructions. Amplification was performed with cycles of 97 °C for 30 sec, 58 °C for 30 sec, and 72 °C for 1 min in a thermal cycler (Takara PCR Thermal Cycler Dice). PCR cycles were 35 for Sox2, Nanog and 28 for β-actin. RT-PCR analysis was performed with the following primers: Sox2 (forward: 5'-CCAAGATGCACAACTCGGAGATCAGC, reverse: 5'- CGAGCCGTTCATGTAGGTCTGCGAG), Nanog (forward: 5'- AGTCCCAAAGGCAAACAACCCACTTC, reverse: 5'- GACTGGCTGTTCTGGGTCTGGTTG), β-actin (forward: 5'-CCCATGCCA TCCTGCGTCTG, reverse: 5'-CGTCATACTCCTGCTTG CTG).

### Immunoblot analysis

Cells were washed with ice-cold PBS and lysed in RIPA buffer [10 mM Tris-HCl (pH 7.4), 0.1% SDS, 0.1% sodium deoxycholate, 1% NP-40, 150 mM NaCl, 1 mM EDTA, 1.5 mM Na_3_VO_4_, 10 mM NaF, 10 mM sodium pyrophosphate, 10 mM sodium β-glycerophosphate and 1% protease inhibitor cocktail set III (Calbiochem)]. After centrifugation for 10 min at 14,000 × g at 4 °C, the supernatants were recovered as the cell lysates, and the protein concentration of the cell lysates was determined by the BCA protein assay kit (Pierce Biotechnology, Inc., Rockford, IL, USA). Cell lysates containing equal amounts of protein were separated by SDS-PAGE and transferred to a polyvinylidene difluoride membrane. The membrane was probed with a primary antibody and then with an appropriate HRP-conjugated secondary antibody according to the protocol recommended by the manufacturer of each antibody. Immunoreactive bands were visualized using Immobilon Western Chemiluminescent HRP Substrate (Millipore, Billerica, MA, USA).

### Mouse studies

Mouse xenograft studies were carried out essentially as previously described. Five- to 8-week-old male BALB/cAJcl-*nu/nu* mice (Clea Japan, Inc.) were, after being anesthetized with avertin (0.375 g/kg intraperitoneally), implanted subcutaneously in the flank region with cells suspended in 200 μL of PBS. After implantation, the recipient mice were monitored for general health status and the presence of subcutaneous tumors. Tumor volume was determined by measuring tumor diameters (measurement of 2 perpendicular axes of tumors) using a caliper and calculated as 1/2 × (larger diameter) × (smaller diameter)^2^. For serial transplantation, primary tumors treated as in the figure legend were excised and, after being washed in chilled sterile PBS, transferred into DMEM/F12, minced with scissors, and incubated in a dissociation enzyme solution (20 mM HEPES-PBS [pH7.4] containing 0.02% DNase I [Sigma, A4572] and 0.02% collagenase II [Sigma, C6885]) for 30 min at 37 °C. After being rinsed with DMEM/F12, the cells were re-suspended in DMEM/F12 and filtered through a 70-μm strainer. After determination of cell number and viability, the single-cell suspension of the tumor cells was subjected to subcutaneous injection. Some of the secondary tumors were excised for photographic documentation or for flow cytometric analysis after being dissociated just as described above for the primary tumors. For systemic administration of SP600125, the SP600125 stock solution (50 mM in DMSO) was diluted in PBS to prepare 200 μL solutions of SP600125 for each injection. The SP600125 solutions were injected intraperitoneally into nude mice. Control mice received 200 μL of DMSO diluted in PBS. Note that all the control- and SP600125-treated mice received an equal volume of DMSO per body weight (3.6 mL/kg/day). All animal experiments were performed under a protocol approved by the Animal Research Committee of Yamagata University.

### Statistical analysis

Results are expressed as the means and standard deviation (SD), and differences were compared using the 2-tailed Student's *t*-test. *P*-values < 0.05 were considered statistically significant and indicated with asterisks in the figures.

## SUPPLEMENTARY MATERIAL AND FIGURES


