# Supplementary material for: Targeting the K-Ras - JNK axis eliminates cancer stem-like cells and prevents pancreatic tumor formation
Source: Oncotarget. 2014 Jun 10;5(13):5100–12. doi: 10.18632/oncotarget.2087 (PMC4148125; doi:10.18632/oncotarget.2087)
Supplement: Supplementary file 1 [file oncotarget-05-5100-s001.pdf]

Targeting the K-Ras - JNK axis eliminates cancer stem-like cells and prevents pancreatic tumor formation

Supplementary Material

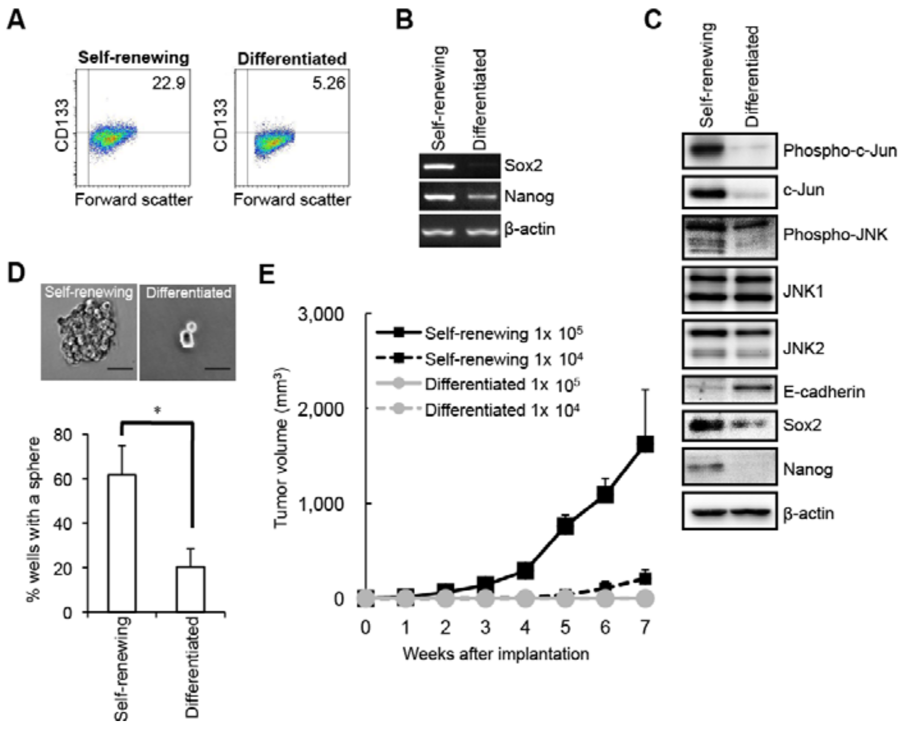

Supplementary Figure S1: Characterization of the PANC-1 CSLC. (A) PANC-1 CSLCs maintained under the stem cell culture condition (Self-renewing) and induced to undergo differentiation in the presence of serum (Differentiated) were subjected to flow cytometric analysis for the cell surface expression of CD133. Representative flow cytometric plots together with the percentages of CD133-positive cells are shown. (B and C) Self-renewing and differentiated PANC-1 CSLCs were subjected to RT-PCR (B) and immunoblot (C) analyses of the indicated mRNAs or proteins. (D) Self-renewing and differentiated PANC-1 CSLCs were assessed for their sphere forming ability by the sphere formation assay. The graph shows the percentage of wells in which a

tumorsphere has been formed from a single cell, and the data represent means + SD from 3 independent experiments. (E) Tumor volumes were measured at the indicated time points after subcutaneous implantation of the indicated numbers of self-renewing and differentiated PANC-1 CSLCs (3 mice for each group). The results are presented in the graph as the means + SD.

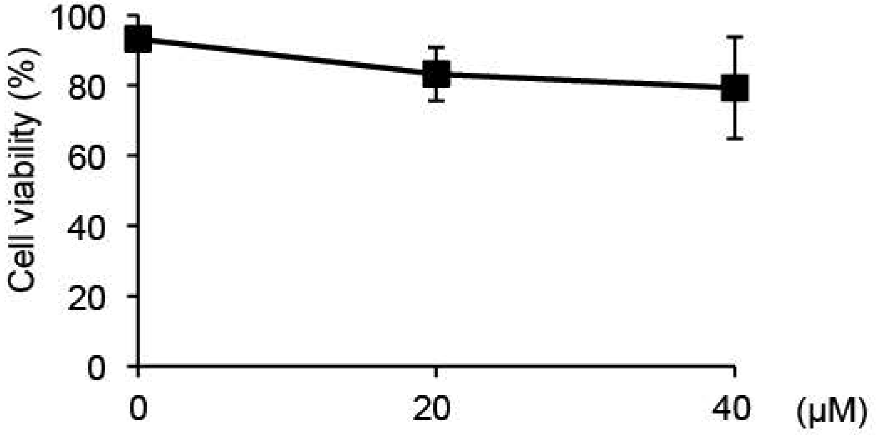

Supplementary Figure S2: The effect of SP600125 on the viability of PANC-1 CSLCs. PANC-1 CSLCs were cultured in the presence of the indicated concentrations of SP600125 for 6 days, when the cells were subjected to determination of the cell viability by dye exclusion. The values in the graphs represent means ± SD of 3 independent experiments.

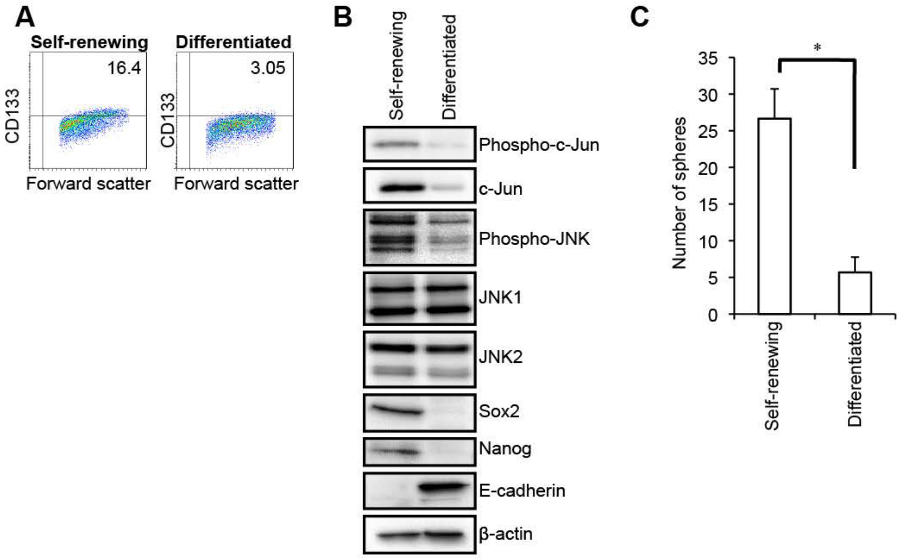

Supplementary Figure S3: Characterization of the PSN-1 CSLC. (A) PSN-1 CSLCs maintained under the stem cell culture condition (Self-renewing) and induced to undergo differentiation in the presence of serum (Differentiated) were subjected to flow cytometric analysis for the cell surface expression of CD133. Representative flow cytometric plots together with the percentages of CD133-positive cells are shown. (B) Self-renewing and differentiated PSN-1 CSLCs were subjected to immunoblot analysis of the indicated proteins to examine the status of the JNK pathway and stem/differentiation marker expression. (C) Self-renewing and differentiated PSN-1 CSLCs were assessed for their sphere forming ability by the sphere formation assay. The graph shows the numbers of spheres formed in each well, and the data represent means + SD from 3 independent experiments.

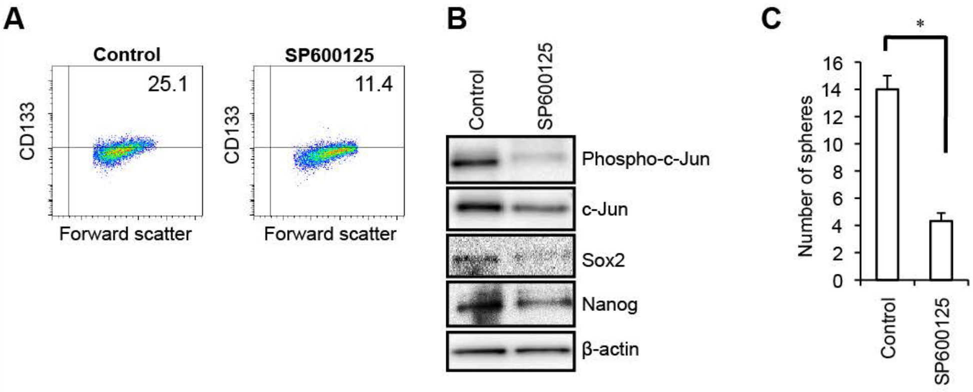

Supplementary Figure S4: Pharmacological inhibition of JNK by SP600125 causes loss of the self-renewal capacity in PSN-1 CSLCs. (A) PSN-1 CSLCs cultured in the absence (Control) or presence of 20 μM SP600125 for 6 days were subjected to flow cytometric analysis for the cell surface expression of CD133. Representative flow cytometric plots together with the percentages of CD133-positive cells are shown. (B) PSN-1 CSLCs cultured as in (A) were subjected to immunoblot analysis of the indicated proteins. (C) PSN-1 CSLCs cultured as in (A) were subjected, after washout of the inhibitor, to the sphere formation assay in the absence of SP600125. The graph shows the numbers of spheres formed in each well, and the data represent means + SD from 3 independent experiments.

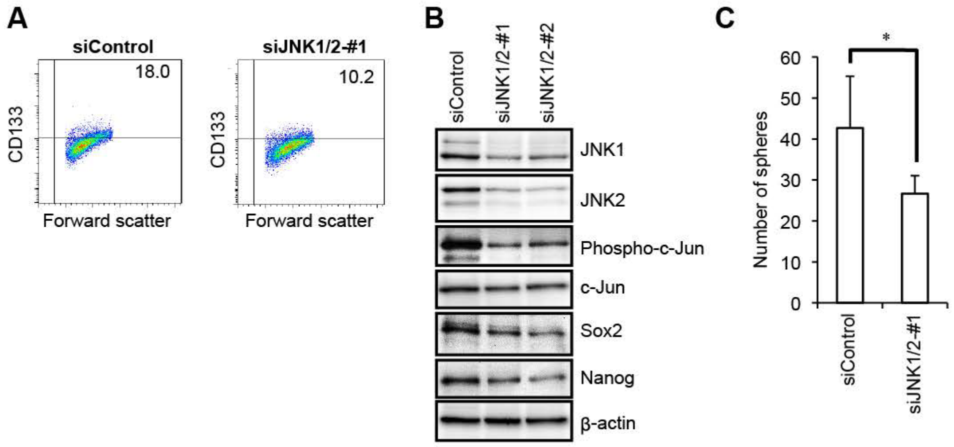

Supplementary Figure S5: Genetic silencing of JNK by siRNA causes loss of the self-renewal capacity in PSN-1 CSLCs. (A) PSN-1 CSLCs were transiently transfected with the indicated combination of siRNAs against JNK1 and JNK2 (siJNK1/2) or with a control siRNA (siControl), as detailed in Materials and methods. After 8 days, the transfected cells were subjected to flow cytometric analysis for the cell surface expression of CD133. Representative flow cytometric plots together with the percentages of CD133-positive cells are shown. (B) PSN-1 CSLCs transiently transfected with the indicated combinations of siRNAs against JNK1 and JNK2 or with a control siRNA were subjected to immunoblot analysis for the expression of the indicated proteins, at 8 days after transfection. (C) PSN-1 CSLCs treated as in (A) were subjected to the sphere formation assay. The graph shows the numbers of spheres formed in each well, and the data represent means + SD from 3 independent experiments.

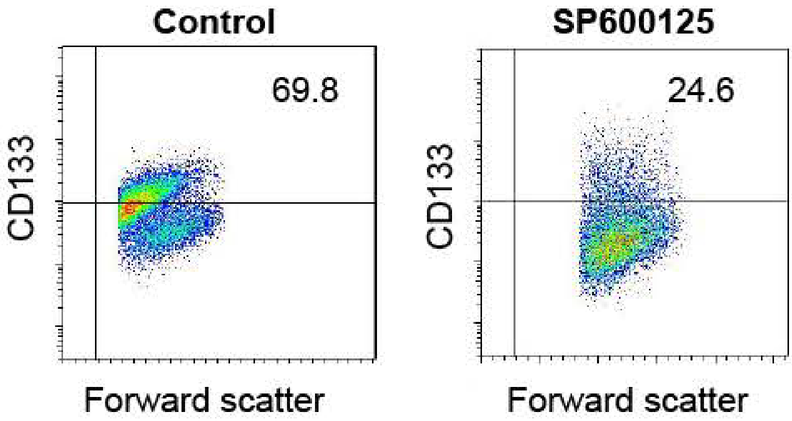

Supplementary Figure S6: Transient JNK inhibition in primary tumors has a sustained inhibitory effect on the CD133 expression of secondary tumor cells. Subcutaneous secondary tumors formed by transplantation of $2x10^6$ primary tumor cells in Figure 3 were excised at the end of observation and fixed after enzymatic dissociation. The secondary tumor cells were then analyzed by flow cytometry for cell surface CD133 expression. The flow cytometric plots are indicated together with the percentages of CD133 positive cells.

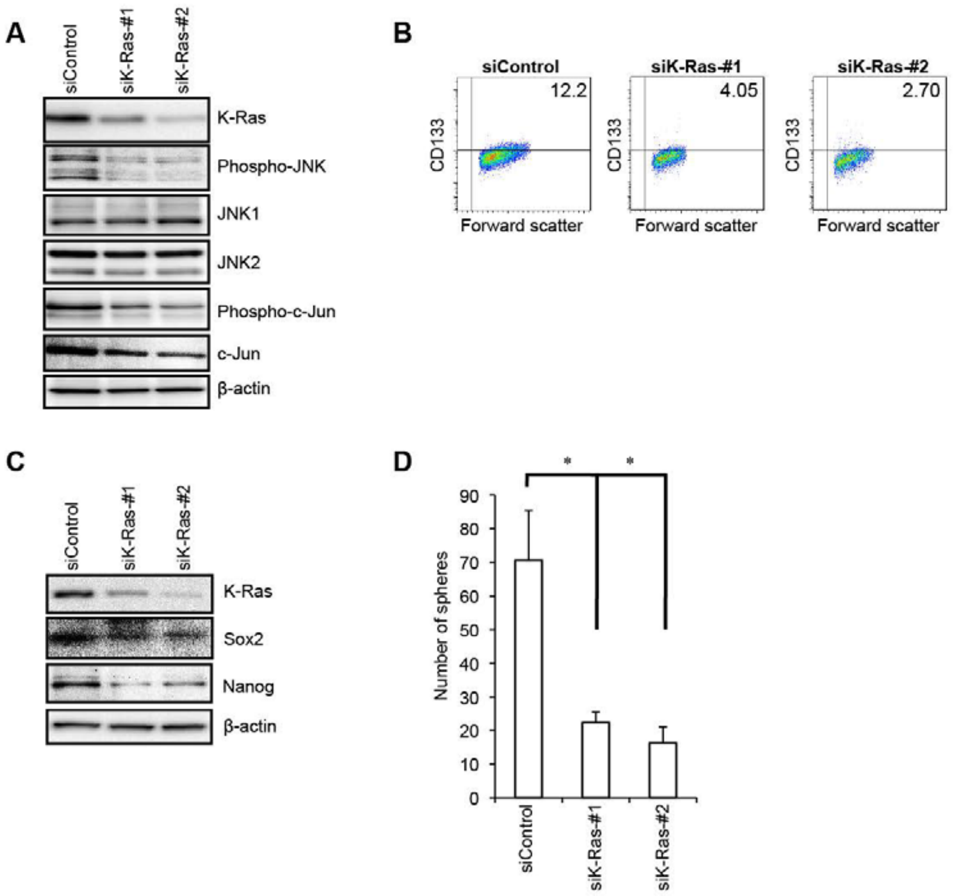

Supplementary Figure S7: K-Ras is required for the maintenance of the JNK pathway activity as well as for the maintenance of the self-renewal capacity of PSN-1 CSLCs. (A) PSN-1 CSLCs transiently transfected with siRNAs against K-Ras (siK-Ras) or with a control siRNA (siControl) were subjected to immunoblot analysis of the indicated proteins at 4 days after transfection, to monitor K-Ras expression as well as the status of the JNK pathway. (B) PSN-1 CSLCs transiently transfected with siRNAs against K-Ras (siK-Ras) or with a control siRNA (siControl) for 8 days were subjected to flow cytometric analysis to examine the cell surface expression of CD133. Representative flow cytometric plots and the percentages of CD133-positive cells are shown. (C) PSN-1 CSLCs treated as in (B) were subjected to immunoblot analysis for the expression of K-Ras and the indicated stem cell markers. (D)

PSN-1 CSLCs treated as in (B) were subjected to the sphere formation assay. The graph shows the numbers of spheres formed in each well, and the data represent means + SD from 3 independent experiments.

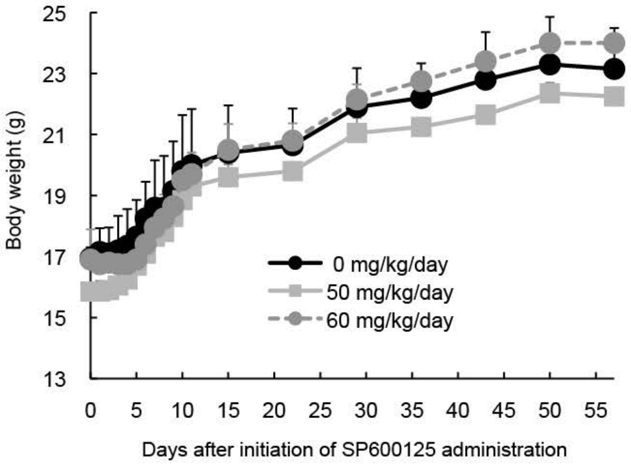

Supplementary Figure S8: Effect of systemic SP600125 administration on mouse body weight. Three groups of mice (2 per each group) were treated with daily intraperitoneal administration of the indicated doses of SP600125 (0, 50, and 60 mg/kg/day) for 10 consecutive days, and their body weight was monitored at the indicated time points. Values represent mean + SD of 2 mice of each group.
